# Supplementary material for: Risk and rates of hospitalisation in young children: a prospective study of a South African birth cohort
Source: medRxiv. 2023 Jun 12:2023.06.08.23289961. Preprint. [Version 1] doi: 10.1101/2023.06.08.23289961 (PMC10312830; doi:10.1101/2023.06.08.23289961)
Supplement: Supplement 1 [file media-1.pdf]

Supplementary Information

Risk and rates of hospitalisation in young children: a prospective study of a South African birth cohort

Catherine J Wedderburn, Julia Bondar, Marilyn T Lake,

Raymond Nhapi, Whitney Barnett, Mark P Nicol, Liz Goddard, Heather J Zar

This PDF file includes:

Supplementary Figure 1: DAG model

Supplementary Figure 2. Causes of hospitalisation stratified by HIV exposure and age groups

Supplementary Table 1: Incidence of hospitalisations in the first 2 years of life stratified by age

Supplementary Table 2: Proportion of LRTI and RSV-LRTI hospitalisations by age category

Supplementary Table 3: Incidence of hospitalisations in the first 2 years of life by HIV exposure status excluding recurrent events

Supplementary Table 4: Incidence of hospitalisations in the first 2 years of life by HIV exposure status

Supplementary Table 5: Impact of birth, breastfeeding, and HIV-related factors on hospitalisation, stratified by HIV status and age

Supplementary Table 6: Association between malnutrition and hospitalisation

Supplementary Figure 1: DAG model
Directed Acyclic Graph (DAG) constructed in www.dagitty.net: HIV exposure and child hospitalisation. This DAG was constructed to examine for possible confounding in the relationship between HIV exposure and child hospitalisation from 0 -24 months in the Drakenstein Child Health Study, South Africa.

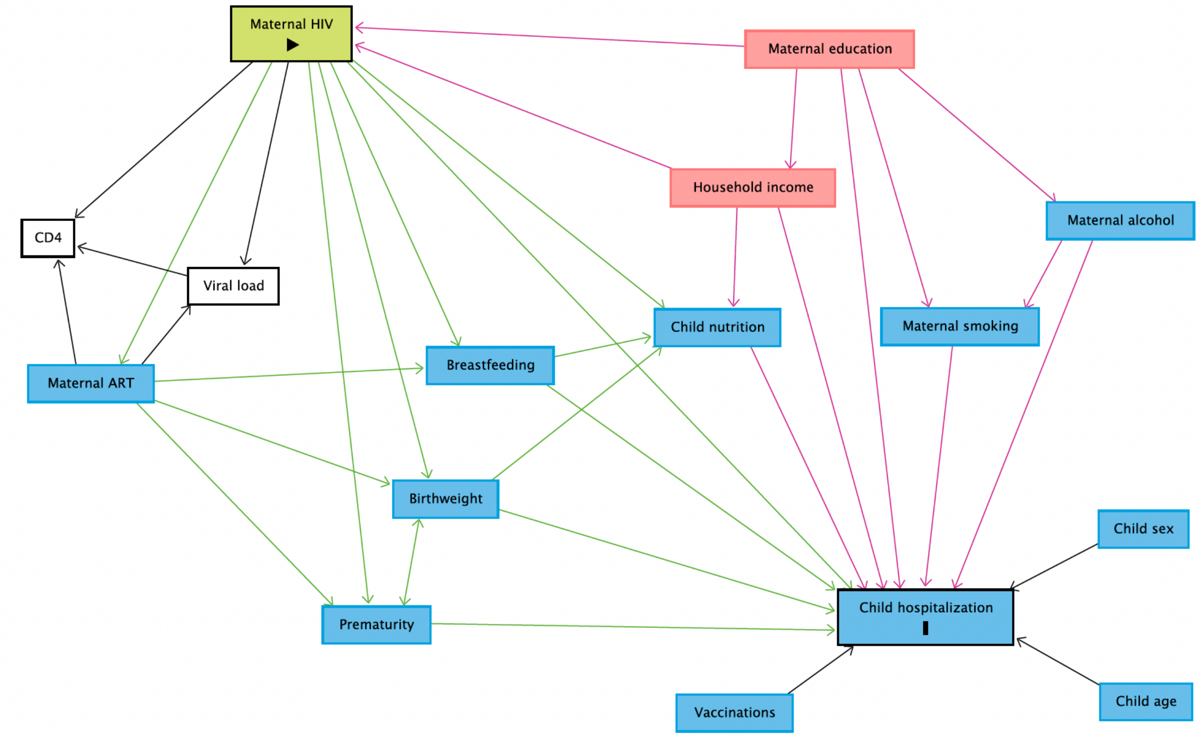

Footnote: Minimal sufficient adjustment sets for estimating the total effect of maternal HIV exposure on child hospitalisation include: Household income and maternal education.

Legend

- exposure
- outcome
- ancestor of exposure
- ancestor of outcome
- ancestor of exposure *and* outcome
- adjusted variable
- unobserved (latent)
- other variable
- causal path
- biasing path

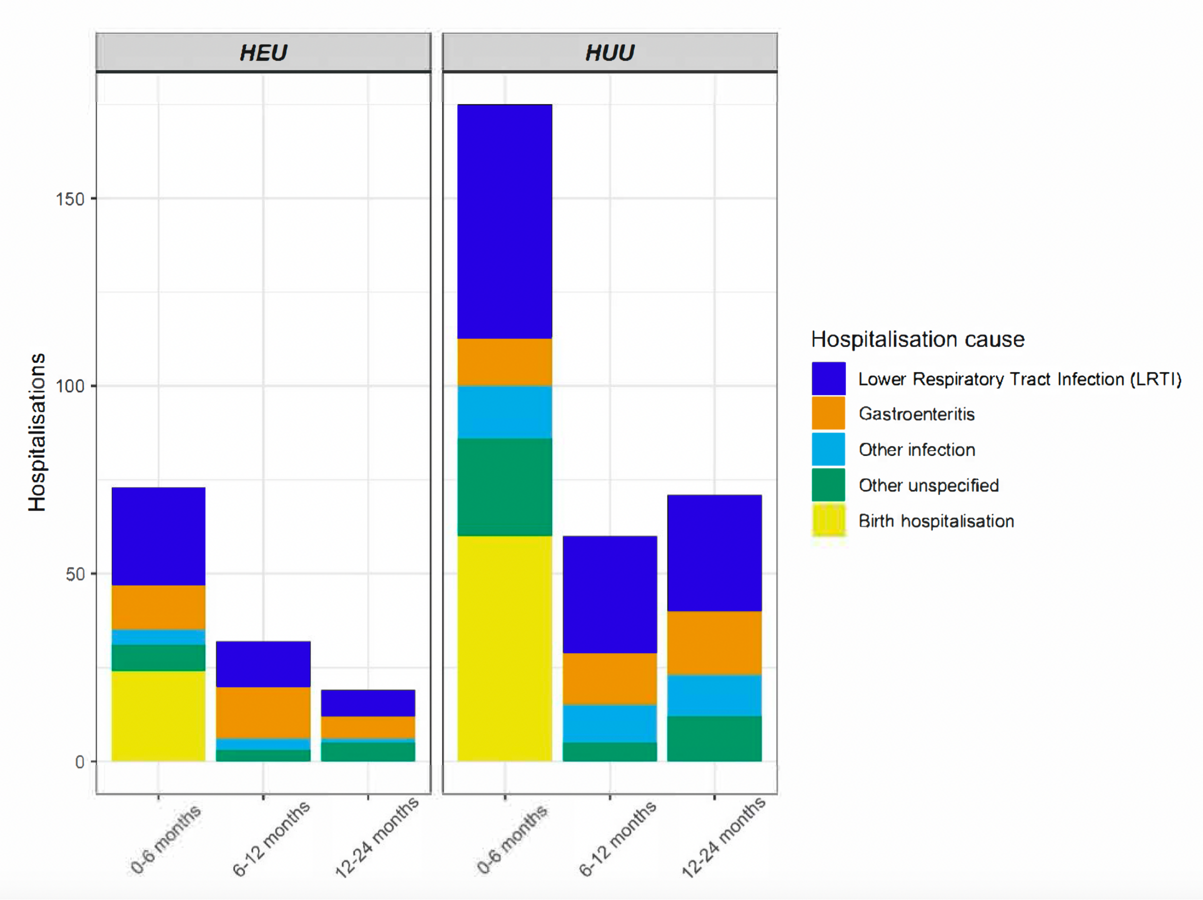

Supplementary Figure 2. Causes of hospitalisation stratified by HIV exposure and age groups Other infections include: meningitis, sepsis, otitis media, urinary tract infection. Other unspecified causes include: Accident and trauma, burns, seizures, nutritional issues (failure to thrive, protein energy malnutrition, micronutrient deficiencies including anaemia)

Supplementary Table 1: Incidence of hospitalisation in the first 2 years of life stratified by age

	**IR / 1000 person years (95% CI)**	**IRR (95% CI)**
All hospitalisations		
0-12 months	314 (281-349)	*0-12 v 12-24 months:* 3.81 (2.99-4.85) *
0-6 months	454 (399-514)	*0-6 v 6-12 months*: 2.69 (2.11-3.44) *
6-12 months	168 (135-207)	*6-12 v 12-24 months:* 2.04 (1.51-2.76) *
12-24 months	82 (65-102)	*0-6 v 12-24 months*: 5.51 (4.30-7.70) *

Footnote: IR = Incidence rate; IRR = Incidence rate ratio; HR = Hazard ratio. *p-value < 0.001

Supplementary Table 2: Proportion of LRTI and RSV-LRTI hospitalisations by age category

	**LRTI hospitalizations (n; % of total LRTI hospitalisations [n=169])**	**RS -LRTI (n; % of total RSV hospitalisations [n=53])**	**LRTI hospitalisations (n; % of hospitalisations per age group)**	**RSV-LRTI (n; % of hospitalisations per age group)**
**Age category**				
**0-12 months**	131/169 (78%)	46/53 (87%)	131/256 (51%)	46/256 (18%)
**0-6 months**	88/169 (52%)	36/53 (68%)	88/164 (54%)	36/164 (22%)
**6-12 months**	43/169 (25%)	10/53 (19%)	43/92 (47%)	10/92 (11%)
**12-24 months**	38/169 (22%)	7/53 (13%)	38/90 (42%)	7/90 (8%)

Footnote: Numbers exclude birth hospitalisations. Abbreviations: LRTI: Lower respiratory tract infection; RSV: Respiratory syncytial virus

Supplementary Table 3: Incidence of hospitalisations in the first 2 years of life by HIV exposure status excluding recurrent events

	HEU IR / 1000 person years	HUU IR /1000 person years	IRR (95% CI)
All hospitalisations			
0-12 months	334 (262-418)	223 (192-258)	1.49 (1.14-1.95) **
0-6 months	510 (388-658)	349 (295-410)	1.46 (1.08-1.97) *
6-12 months	144 (82-234)	89 (62-123)	1.62 (0.90-2.92)
12-24 months	50 (25-90)	45 (31-62)	1.12 (0.57-2.20)

Footnote: ** p-value < 0.01; * p-value < 0.05. Abbreviations: HEU = HIV-exposed uninfected; HUU = HIV-unexposed uninfected; IR = Incidence rate; IRR = Incidence rate ratio

Supplementary Table 4: Incidence of hospitalisations in the first 2 years of life by HIV exposure status

				Unadjusted	*Adjusted model 1*	*Adjusted model 2*
	**All IR / 1000 person years**	**HEU IR / 1000 person years**	**HUU IR /1000 person years**	**IRR (95% CI)**	**HR (95% CI)**	**HR (95% CI)**
0-12 months	235 (207-266)	346 (274-431)	205 (175-238)	1.69 (1.30-2.21) ***	1.53 (1.14-2.07) **	1.59 (1.16-2.18) **
0-6 months	299 (255-349)	424 (314-559)	265 (219-319)	1.60 (1.14-2.23) **	1.48 (1.03-2.12) *	1.56 (1.07-2.28) *
6-12 months	168 (135-207)	265 (179-379)	142 (107-183)	1.87 (1.20-2.91) **	1.73 (1.05-2.85) *	1.76 (1.00-3.08) *
12-24 months	82 (65-102)	77 (45-124)	84 (65-106)	0.92 (0.54-1.57)	0.91 (0.53-1.55)	0.78 (0.44-1.38)

Footnote: Unadjusted incident rate ratios and adjusted hazard models for hospitalisations in the first 2 years of life by HIV exposure excluding birth hospitalisations.
IR = Incidence rate; IRR = Incidence rate ratio; HR = Hazard ratio. Multivariate models adjusted for (1) maternal education and household income; (2) maternal education, household income, maternal age at birth and maternal smoking. *** p-value < 0.001; ** p-value < 0.01; * p-value < 0.05
Abbreviations: HR = Hazard ratio; HEU = HIV-exposed uninfected; HUU = HIV-unexposed uninfected; IR = Incidence rate; IRR = Incidence rate ratio; OR = Odds ratio.

Supplementary Table 5: Impact of birth, breastfeeding and HIV-related factors on hospitalisations stratified by HIV status and age

		HEU<br>HR (95% CI)	HUU<br>HR (95% CI)	Total<br>HR (95% CI)
Birth factors				
Prematurity				
0-12 months		1.94 (1.11-3.37) *	1.75 (1.22-2.52) **	1.85 (1.36-2.52) ***
	0-6 months	1.67 (0.89-3.13)	2.04 (1.34-3.11) ***	1.95 (1.37-2.78) ***
	6-12 months	2.66 (1.07-6.62) *	1.26 (0.66-2.41)	1.74 (0.99-3.04)
12-24 months		1.48 (0.43-5.11)	0.97 (0.45-2.06)	1.07 (0.56-2.04)
Feeding				
Ever breastfed				
0-12 months		0.85 (0.51-1.43)	0.76 (0.42-1.39)	0.63 (0.46-0.87) **
12-24 months		1.00 (0.39-2.62)	1.07 (0.35-3.22)	1.08 (0.60-1.94)
Breastfeeding for a year				
0-12 months		1.02 (0.39-2.62)	0.71 (0.50-1.02)	0.67 (0.48 -0.93) *
12-24 months		0.97 (0.24-3.88)	0.64 (0.36-1.11)	0.70 (0.42 -1.16)
Immunization timing by 9 months				
0-12 months		1.48 (0.87-2.52)	1.36 (0.96-1.92)	1.39 (1.04-1.87) *
12-24 months		1.34 (0.52-3.48)	1.03 (0.60-1.77)	1.09 (0.68-1.75)
HIV-related variables				
CD4 - categorical (>500 vs <=500)				
0-12 months		0.99 (0.56-1.76)	-	-
	0-6 months	0.92 (0.46-1.87)	-	-
	6-12 months	1.11 (0.43-2.83)	-	-
12-24 months		0.88 (0.33-2.35)	-	-
Viral load-categorical (>= 40 vs <40)				
0-12 months		1.51 (0.79-2.86)	-	-
	0-6 months	0.92 (0.43-1.98)	-	-
	6-12 months	4.43 (1.52-12.87) **	-	-
12-24 months		1.31 (0.40-4.30)	-	-
ART regimen initiation (Before vs during pregnancy)				
0-12 months		0.86 (0.51-1.44)	-	-
	0-6 months	1.06 (0.58-1.93)	-	-
	6-12 months	0.54 (0.20-1.40)	-	-
12-24 months		0.76 (0.29-1.99)	-	-

Footnote: Results with birth hospitalisations excluded. *** p-value < 0.001; ** p-value < 0.01; * p-value < 0.05 for unadjusted models. Definitions: Prematurity (<37 vs >= 37 weeks); Ever breastfeeding (>0 months vs = 0 months); Breastfeeding for a year (>11 months vs <= 11 months). Abbreviations: ART = antiretroviral therapy; HR = Hazard ratio; HEU = HIV-exposed uninfected; HUU = HIV-unexposed uninfected

Supplementary Table 6: Association between malnutrition and hospitalisation

	Underweight			
	**Total**	**HEU**	**HUU**	**OR (95% CI)**
0-12 months	47 (19%)	19 (24%)	28 (17%)	1.62 (0.83-3.12)
0-6 months	35 (22%)	14 (29%)	21 (19%)	1.76 (0.80-3.85)
6-12 months	12 (14%)	5 (17%)	7 (12%)	1.46 (0.40-5.03)
12-24 months	9 (11%)	4 (25%)	5 (7.6%)	4.07 (0.90-17.69)
	**Stunted**			
0-12 months	71 (31%)	27 (38%)	44 (28%)	1.63 (0.90-2.94)
0-6 months	47 (32%)	16 (38%)	31 (30%)	1.45 (0.68-3.06)
6-12 months	24 (28%)	11 (38%)	13 (23%)	2.07 (0.78-5.51)
12-24 months	14 (18%)	4 (27%)	10 (16%)	2.41 (0.65-8.32)
	**Wasted**			
0-12 months	29 (15%)	11 (19%)	18 (14%)	1.50 (0.64-3.37)
0-6 months	18 (17%)	8 (28%)	10 (13%)	2.51 (0.86-7.22)
6-12 months	11 (13%)	3 (10%)	8 (14%)	0.71 (0.15-2.68)
12-24 months	8 (10%)	3 (20%)	5 (7.9%)	2.90 (0.54-13.55)

Abbreviations: HEU = HIV-exposed uninfected; HUU = HIV-unexposed uninfected; OR = Odds ratio
